# Effect of long term CPAP therapy on cardiac parameters assessed with cardiac MRI

**DOI:** 10.1007/s10554-020-02024-y

**Published:** 2020-09-14

**Authors:** W. Wuest, M. S. May, M. Wiesmueller, M. Uder, A. Schmid

**Affiliations:** Radioloical Institute, Friedrich-Alexander-University-Erlangen-Nuremberg, Maximiliansplatz 1, 91054 Erlangen, Germany

**Keywords:** Cardiac MRI, Sleep apnea, CPAP therapy, Follow up

## Abstract

The obstructive sleep apnoea syndrome (OSAS) is a disorder with a high prevalence and is associated with an elevated cardiovascular risk and increased morbidity and mortality. For longitudinal studies and functional analysis cardiac MRI is regarded as the gold standard. Aim of this study was to evaluate the long-term effect of continuous positive airway pressure (CPAP) therapy on cardiac functional parameters with cardiac Magnetic Resonance Imaging (cMRI). 54 patients with OSAS (mean apnea hypopnea index-AHI: 31) were prospectively enrolled in this study and cMRI was performed before and after 7 months of CPAP therapy. Data were acquired on a 1.5 T MRI and right and left ventricular cardiac function were analysed. CPAP treatment was considered compliant when used* ≥ *4 h per night. 24-h blood pressure was measured at baseline and follow up. 33 patients could be assigned to the compliance group. Left ventricular stroke volume (LV SV) and right ventricular ejection fraction (RV EF) improved significantly with CPAP therapy (LV SV from 93 ± 19 to 99 ± 20 ml, p = 0.02; RV EF from 50 ± 6 to 52 ± 6%, p = 0.04). All other cardiac parameters did not change significantly while mean systolic and diastolic blood pressure improved significantly (p < 0.01). 21 patients were assigned to the non-compliance group and were considered as a control group. There were no relevant differences in cardiac parameters between baseline and follow up examination in these patients. CPAP therapy seems to improve LV SV, RV EF, systolic and diastolic blood pressure in OSAS patients, but reproducibility of our results need to be confirmed in a larger patient population.

## Introduction

Obstructive sleep apnea syndrome (OSAS) is a common sleep-related breathing disorder associated with heart failure [1–3]. People with OSAS suffer from complete closure of the pharynx, gasping episodes, sleep fragmentation, and daytime sleepiness, which could lead to hypoxia of the heart, elevated intrathoracic pressure, increased left ventricular afterload, and acute hypertension provoked by bursts of sympathetic activity [4–8]. The prevalence of OSAS is estimated to be at least 4% in men and 2% in women [9]. The standard treatment is continuous positive airway pressure (CPAP), first described by Sullivan et al. [4] in 1981. CPAP has been demonstrated to improve daytime performance and reduce cardiovascular effects associated with OSAS [10, 11]. Cardiac morphology and function are affected by OSAS, yet the exact mechanisms are still not completely understood. Most studies assessing cardiac morphology and function in OSAS have been performed with echocardiography, mainly focusing on the left ventricle. The quantitative assessment of right ventricular size and function is however often difficult. Generally, cardiac magnetic resonance imaging (cMRI) is considered the gold standard for volumes, EF, and mass and is well suited for the determination of left ventricular (LV) and right ventricular (RV) parameters because of the 3-dimensional volume acquisition [12–14].

The purpose of this study was to investigate the long-term effects of CPAP therapy on cardiac morphology and function in OSAS using cMRI and the long-term effect on blood pressure.

## Materials and methods

### Patient population

54 (44 males, 10 females) patients were included in this prospective study. Patients with atrial fibrillation, moderate to severe valvular heart disease, known coronary artery disease (CAD), previous cardiac surgery or any contraindication to cMRI or CPAP were excluded from the study.

All study subjects underwent a complete medical work-up including medication, clinical chemistry and physical examination. Use of medication was assessed at baseline and at follow-up measurements. Before OSAS treatment diagnostic polysomnography was performed. In this setting, the degree of OSAS and the sleep disturbance was quantified. The apnea hypopnea index (AHI) was assessed. Sleep stages were scored according to the standard criteria of the American Academy of Sleep Medicine [15]. Obstructive apneas were defined as the absence of oronasal flow for at least 10 s. Hypopneas were defined as reduction in airflow to ≤ 50% of the preceding stable baseline for 10 s or longer together with a dip in oxygen saturation ≥ 4%. The mean number of apneas and hypopneas per hour of sleep was calculated as AHI.

Only in CPAP-naive patients newly diagnosed with OSAS based on the overnight polysomnographic (PSG) exam (AHI > 5/h) baseline cMRI and 24 h blood pressure monitoring was performed.

After baseline cMRI all patients initiated CPAP treatment with CPAP machines equipped with compliance monitors that measured CPAP use. The prescribed CPAP pressure was based on a titration study that eliminated respiratory events and improved oxyhemoglobin saturation during sleep. Adequate compliance to the prescribed pressure of CPAP was defined as more than 4 h of CPAP use per night on a routine basis [16, 17]. Patients with usage of CPAP therapy for less than 4 h were enrolled in the control group (non-compliance group). All patients were evaluated at baseline and after 7 months of CPAP therapy.

Written informed consent was obtained from all patients and the institutional review board approved the study.

### Image analysis

CMRI analysis was an average of independent readings performed by two observers (W.W. and A.S.) with more than 10 and 20 years of experience in cMRI, respectively, who were blinded to patient and other imaging data. Only patients examined at baseline and after 7 months of CPAP therapy were included in the final analysis. All patients were examined with a 1.5 T scanner (Magnetom Espree Quantum, Siemens Healthineers, Erlangen, Germany) and a 4-channel phased array body coil in supine position. Short, long axis and axial cine imaging was performed during breath-hold employing segmented 2D spoiled GRE sequences (field of view 240 × 320 mm^2^, matrix 126 × 256, repetition time 9.9 ms, echo time 4.8 ms, flip angle 30°, slice thickness 8 mm) with a temporal resolution of 45 ms per image phase.

**Fig. 1:**
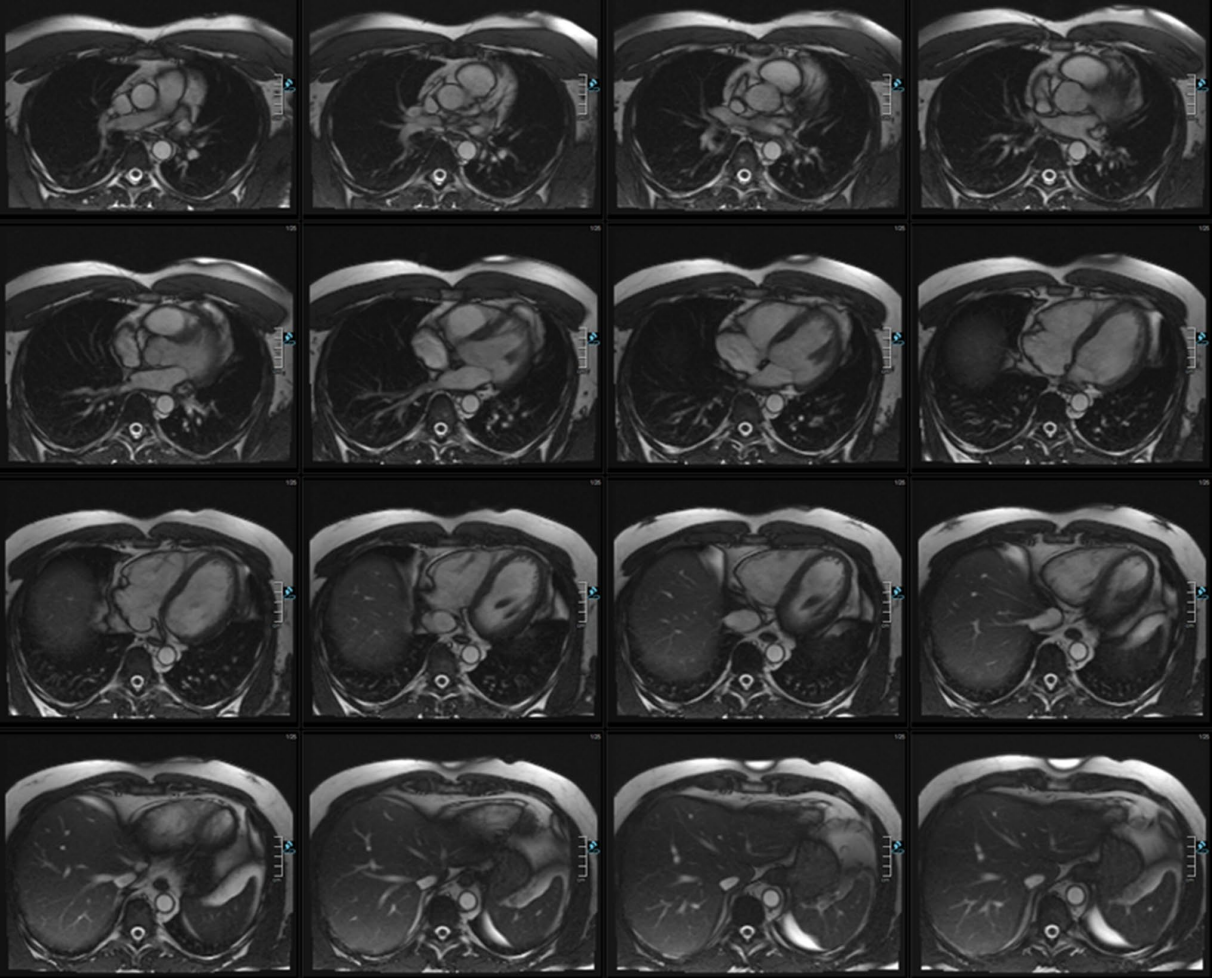
Representative example of an axial stack used for evaluation of right ventricular parameters

**Fig. 2:**
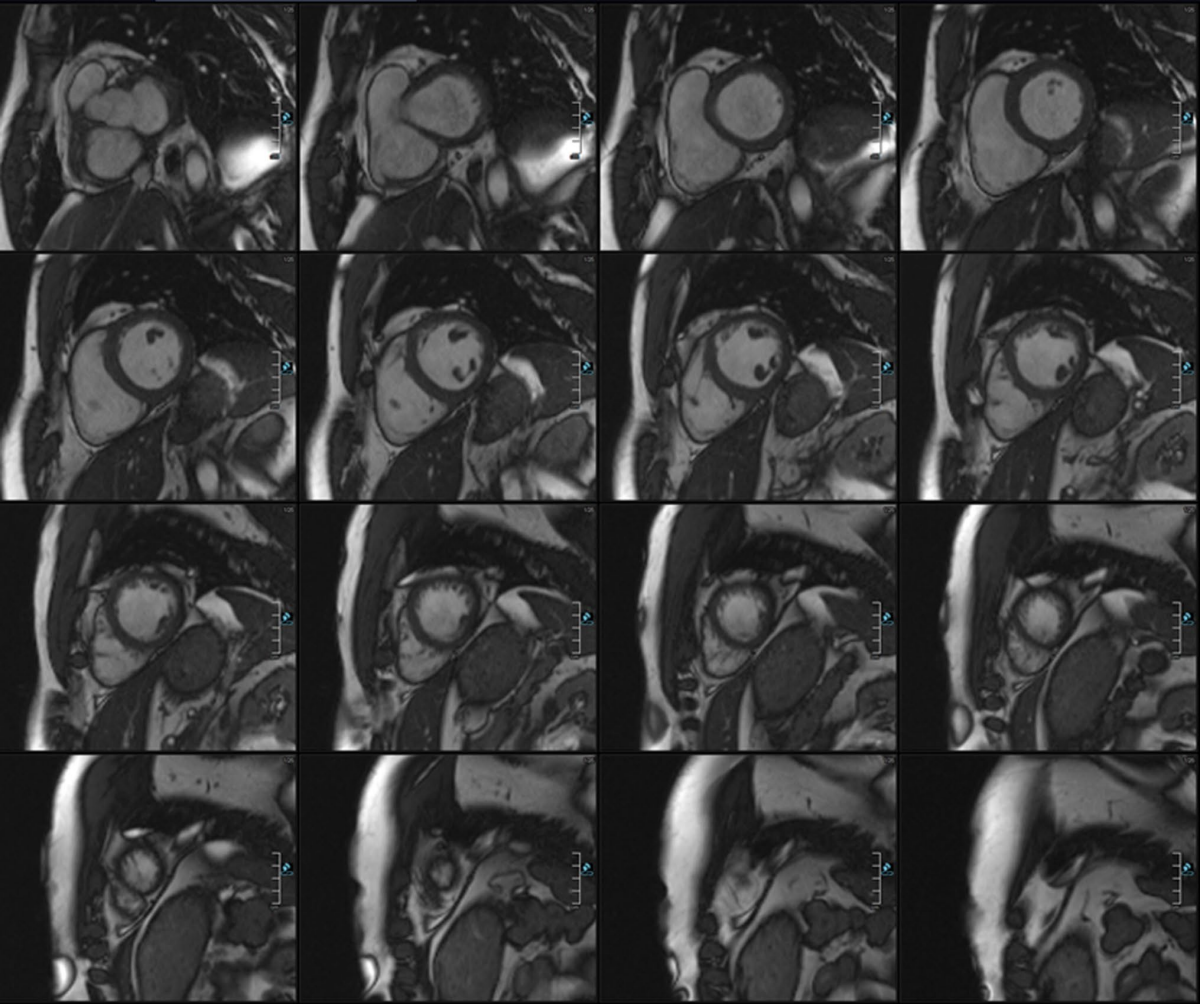
Representative example of a short axis stack used for evaluation of left ventricular parameters

Figures 1 and 2 show examples of representative short axis stack for left ventricular and axial stack for right ventricular evaluation. LV endo- and epicardial and RV endocardial borders of each section from the base to the apex were identified manually at end diastole and end systole because manual delineation has been demonstrated to be the most accurate method for cardiac function analysis with MR imaging [18]. LV global mass and LV functional parameters were analysed in the short axis and RV functional parameters in the axial slices by using dedicated software (ARGUS; Siemens Healthineers, Erlangen, Germany). The trabecular network and the papillary muscles were attributed to the blood volume. The interventricular septum was regarded as part of the left ventricle with epi- and endocardial marks set as close together as possible.

The following parameters were evaluated: ejection fraction (EF), enddiastolic volume (EDV), endsystolic volume (ESV), stroke volume (SV), and LV myocardial mass (MM).

### Statistical analysis

The nonparametric Wilcoxon’s matched-pairs test was used to evaluate differences between baseline and follow-up examination results. To compare baseline values between the study and control group student`s t-test was used. A p-value of < 0.05 was considered statistical significant. All parameters are given as mean ± standard deviation. Statistical analysis was performed using the software package SPSS Statistics version 21 (SPSS Inc./IBM, Chicago, IL).

## Results

54 patients underwent both baseline and follow-up cMRI and were included in the data analysis. 33 patients (7 females and 26 males, mean age: 51 ± 11) were assigned to the compliance group and 21 patients (3 females, 18 males, mean age: 57 ± 7) to the non-compliant (control) group.

See Tables 1, 2, and 3 for complete patient characteristics.

**Table 1:** Baseline characteristics of total population (n = 54)

	Study group	Control group
Female (n)	7 [21%]	3 [14%]
Age (years)	51 ± 11	57 ± 7
Body-mass-index (kg/m^2^)	33 ± 7	31 ± 5
AHI (h)	34 ± 24 [range 5–103]	27 ± 19 [range 7–79]
Blood pressure (mmHg)		
Systolic	135 ± 12	131 ± 13
Diastolic	83 ± 9	83 ± 10
Hypertonus (> 140/90 mmHg)	9 [27%]	3 [14%]
Asthma	3	0
Dyslipidemia	1	1
Mean score on EES	9 ± 4	11 ± 5
Min. O_2_ (%)	72 ± 10	75 ± 11

**Table 2:** Complete patient characteristics of the study group (n = 33)

ACE-inhibitor/AT1-Bl	β-blocker	ASS	Ca-antagonist (verapa.-typ)	β2 mimetics or theopyllin	Statin	Change of medication	Height (cm)	Weight (kg)	BMI (baseline)	BMI (follow up)
0	0	0	0	0	0	No	172	109	36.8	33.8
0	0	0	0	0	0	No	190	98	27.1	27.4
0	0	0	0	0	0	No	170	99	34.2	33.9
1	1	1	0	0	0	No	157	130	52.8	50.4
0	0	0	0	0	0	No	178	82	25.9	25.9
0	0	0	0	1	0	No	168	90	31.9	31.9
0	0	0	0	0	0	No	187	130	37.2	35.5
0	0	0	1	0	0	No	174	92	30.4	28.4
1	1	1	0	0	0	No	176	80	25.8	25.8
1	1	0	0	0	1	No	164	109	40.5	42.0
0	0	0	0	0	0	No	190	93	25.7	26.3
0	0	0	0	0	0	No	176	73	23.6	24.5
0	0	0	0	0	0	No	182	105	31.7	31.7
0	0	0	0	0	0	No	180	109	33.6	33.6
0	1	0	0	0	1	No	182	101	30.5	31.7
0	0	0	0	0	0	No	165	98	36.0	36.0
0	0	0	0	0	0	Yes	170	106	36.8	36.1
0	0	0	0	0	0	No	180	120	37.0	38.2
0	0	0	0	0	0	No	183	132	39.5	38.0
0	0	0	0	0	0	No	183	112	33.5	32.9
1	0	0	0	0	0	No	155	72	30.0	29.1
0	0	0	0	0	0	No	185	104	30.4	31.2
0	0	0	0	0	0	Yes	173	94	31.4	30.1
0	0	0	0	0	0	No	163	88	33.2	33.2
0	0	0	0	0	0	No	175	73	23.8	23.5
0	0	0	0	0	0	No	184	107	31.6	30.4
0	0	0	0	0	0	No	165	93	34.1	31.2
0	0	0	0	0	0	No	190	160	44.3	44.3
0	0	0	0	0	0	No	174	85	28.1	29.8
0	0	0	0	0	0	No	171	71	24.3	24.6
0	0	0	0	0	0	No	182	124	37.4	31.1
0	0	0	1	1	0	No	170	128	44.4	46.8
0	0	0	0	1	0	No	176	77	24.9	24.9

**Table 3:** Complete patient characteristics of the control group (n = 21)

ACE-inhibitor/AT1-BL	β-blocker	ASS	CA-antagonist (verapa.-typ)	β2-mimetics or theopyllin	Statin	Change of medication	Height (cm)	Weight (kg)	BMI (baseline)	BMI (follow up)
0	0	0	0	0	0	No	170	108	37.5	38.1
0	0	0	0	0	0	No	185	110	32.1	30.4
1	0	0	0	0	0	No	176	89	28.8	27.5
0	0	0	0	0	0	No	185	115	33.6	30.7
1	0	0	0	0	0	No	172	107	36.2	35.9
0	0	0	0	0	0	No	170	83	28.8	28.8
1	0	0	1	0	0	No	172	60	20.3	20.3
0	0	0	0	0	0	No	168	105	37.2	36.8
0	0	0	0	0	0	No	172	103	34.9	34.9
0	0	0	0	0	0	No	156	67	27.5	26.7
0	0	0	0	0	0	No	177	115	36.7	36.7
0	0	0	0	0	0	No	178	123	38.9	37.9
1	0	0	1	0	0	No	168	76	26.9	26.5
0	1	1	0	0	0	No	170	69	23.9	23.6
0	0	0	0	0	0	No	185	128	37.4	38.0
0	0	0	0	0	0	No	184	84	24.8	25.4
0	0	0	0	0	0	No	170	65	22.5	22.5
1	1	0	0	0	0	No	165	89	32.7	34.1
0	0	0	0	0	0	No	180	91	28.0	28.0
0	0	0	0	0	0	No	185	100	29.2	29.2
0	0	0	0	0	0	No	184	94	27.8	27.5

### Blood pressure (24 h), body mass index (BMI) and AHI

#### Compliant group

CPAP therapy was used 5.6 ± 1.2 h/night.

At baseline, the mean BMI was 33 ± 7 kg/m^2^, at follow up 33 ± 6 kg/m^2^. Baseline AHI was 34 ± 24 h, the minimum oxygen saturation was 72 ± 10%.

24 h blood pressure monitoring results could be retrieved in 25/33 (76%) patients.

At time of enrollment, the mean systolic and diastolic BP was 135 ± 12 mmHg and 83 ± 9 mmHg, respectively. At follow up the mean systolic and diastolic BP improved significantly (126 ± 10 mmHg and 76 ± 8 mmHg, p = 0.005 and p = 0.002).

7/33 (21%) patients were on antihypertensive drugs (1 × ACE-inhibitor, 1 × β-blocker). In 2/33 (6%) patients antihypertensive medication was started during CPAP therapy.

#### Non compliant group

CPAP therapy was used 1.9 ± 1.2 h/night.

At baseline, the mean BMI was 31 ± 5 kg/m^2^, at follow up 30 ± 5 kg/m^2^. Baseline AHI was 27 ± 19 h, the minimum oxygen saturation was 75 ± 10%.

24 h blood pressure monitoring results could be retrieved in 13/21 (62%) patients.

At time of enrollment, the mean systolic and diastolic BP was 131 ± 13 mmHg and 83 ± 10 mmHg, respectively. At follow up the mean systolic and diastolic BP improved but only the diastolic pressure significantly (126 ± 14 mmHg and 79 ± 13 mmHg, p = 0.1 and p = 0.04).

6/21 (28%) patients were on antihypertensive drugs and antihypertensive medication was not changed during CPAP therapy.

Baseline AHI and blood pressure results did not differ significantly between the study and non compliant group.

### CMRI

Baseline cardiac parameters did not differ significantly between the study and control group. See Tables 4 and 5 for complete cMRI results.

**Table 4:** Left ventricular (LV) baseline and follow-up results in the study and control group

	Baseline	Follow up	p-values	Baseline	Follow up	p-values
	compliant	compliant		Non-compliant	Non-compliant	
LV mass (g)	135 ± 28	137 ± 28	0.58	135 ± 28	137 ± 28	0.78
dEDV (ml)	149 ± 29	154 ± 29	0.20	147 ± 41	149 ± 37	0.30
ESV (ml)	55 ± 18	55 ± 18	0.95	55 ± 20	58 ± 20	0.29
SV (ml)	93 ± 19	99 ± 20*	0.02*	92 ± 25	92 ± 24	0.93
EF (%)	63 ± 8	64 ± 8	0.29	63 ± 7	62 ± 8	0.49

^*^Significant results are marked

**Table 5:** Right ventricular (RV) baseline and follow-up results in the study and control group

	Baseline	Follow up	p-values	Baseline	Follow up	p-values
	compliant	compliant		Non-compliant	Non-compliant	
EDV (ml)	186 ± 37	188 ± 37	0.80	178 ± 35	179 ± 36	0.82
ESV (ml)	93 ± 26	92 ± 24	0.50	86 ± 16	86 ± 16	0.51
SV (ml)	92 ± 18	92 ± 24	0.18	93 ± 23	93 ± 23	1
EF (%)	50 ± 6	52 ± 6*	0.04*	52 ± 5	51 ± 5	0.44

^*^Significant results are marked

Image quality was diagnostic in all patients and the examination could be completed in all cases.

#### Compliant group

LV SV and RV EF improved significantly after CPAP therapy (93 ± 19 ml vs. 99 ± 20 ml—p = 0.02 and 50 ± 6% vs. 52 ± 6% at follow up—p = 0.04). All other LV and RV cardiac parameters did not change significantly. In 3/33 patients (9%) LV EF improved by 15% and in 1/33 patients (3%) RV EF increased by 15%.

#### Non compliant group

There was no significant difference between baseline and follow up examination in the control group.

## Discussion

We investigated cardiac effects of CPAP-therapy on 54 patients with OSAS after 7 months of treatment. LV SV, RV EF and systolic as well as diastolic blood pressure improved significantly in the compliance group using CPAP masks at least 4 h per night.

In OSAS recurrent episodes of complete obstruction (apnea) or partial obstruction (hypopnea) of the upper airway lead to intermittent hypoxia during sleep. Sleep can be divided in different stages and heart rate and systemic blood pressure are reduced and reach its lowest levels during the early periods of slow-wave sleep [19, 20]. Reasons for lowering of the blood pressure are the recumbent resting situation and sleep itself [21]. The apnea ventilation consists of different phases with a rising heart rate in the immediate postapneic period [22] and the highest blood pressure within the first postapneic breath [23]. By lowering nocturnal hypoxemia CPAP therapy reduces the systemic blood pressure, decreases the nocturnal blood pressure surge and alleviates the prognosis [24, 25]. In our study CPAP therapy reduced systolic and diastolic blood pressure significantly which is well in line with previous results [26]. Interestingly, in the non-compliant group we found no effect of CPAP therapy (< 4 h per night) on cardiac parameters however the diastolic blood pressure decreased significantly. With no change of medication in this group, we can only speculate that usage of CPAP therapy for less than 4 h has a positive effect on blood pressure with the diastolic pressure being the first to improve.

Not only the systemic pressure alters during sleep but also there is evidence that pulmonary artery pressure also changes during the obstructive sleep apnea cycle [27]. The strength of RV contraction is affected by pulmonary arterial resistance and in OSAS repetitive nocturnal elevations in pulmonary artery pressure and increased sympathetic discharges during apneic episodes may lead to RV remodeling and intermittent RV pressure overload [28, 29]. Other reasons for RV dysfunction may be due to chronic impacts of intermittent nocturnal hypoxemia and hypercapnia, LV dysfunction and systemic hypertension. Several studies report a significant fall in pulmonary arterial hypertension after 3 to 6 months of CPAP therapy [30–32]. We could show a significant increase of the right ventricular ejection fraction with no change of the other right ventricular cardiac parameters.

Apart from our study there are only two other previous reports using cMRI to examine the longitudinal effect of CPAP on cardiac functional parameters in OSAS patients.

In the first study, Magalang et al. [33] investigated the effect of CPAP therapy in 13 patients after 3 months of therapy. They found no significant changes in LV functional parameters however, a significant decrease of RV EDV/ESV volumes and a trend towards improved RV EF.

In the second published study by Colish et al. [34] 47 patients were examined with cMRI and echocardiography at baseline and after 12 months of CPAP therapy. They also reported a trend towards increased RV EF but additionally a significantly decreased LV mass.

Some of the previously published studies using echocardiography report a positive effect of CPAP therapy on LV functional parameters associated with OSAS [35], some do not [36]. Bayram et al. [37] treated 20 patients for 6 months with CPAP-therapy and found no change in LV EF. In contrast, Butt et al. [38] report a significant change of LV EF and LV EDV using M-Mode with no difference in LV ESV after treating 37 patients for 6 months with CPAP-therapy. However, in the same study these results were not reproducible using 2D and 3D echocardiography with no difference in LV EF, LV EDV and ESV.

Echocardiography data on the RV is sparse and only some papers are available. Bayram et al. [37] for example included 20 patients with newly diagnosed OSAS in their study. They found RV systolic and diastolic dysfunction improvement 6 months after CPAP therapy assessed by tissue Doppler imaging (TDI).

One reason for discrepancy between the results of cMRI and echocardiography studies could be that MR imaging enables more accurate morphologic analyses than does echocardiography [39]. CMRI is the gold standard for the analysis of both LV and RV functional parameters [14]. This is especially important in patients suffering from OSAS, who are mainly obese, which represents a major limitation for echocardiography to acquire adequate images.

### Limitations

Our study has several limitations.

First, the number of patients included is rather small but within the range of previous reported studies.

Second, not all 24-h blood pressure monitoring results could be retrieved (76% in the compliant and 62% in the non compliant group).

Third, there was a change of medication (1 × ACE-inhibitor, 1 × β-blocker) in 2 patients (study group) between baseline and follow up cMRI. However in 31/33 (94%) patients medication was not altered thus we believe our results are representative for the effect of CPAP therapy.

Fourth, due to the small but significant differences found between RV and LV functional parameters the clinical significance needs to be confirmed in a larger patient cohort.

## Conclusion

Using CPAP therapy > 4 h/night seems to improve LV SV and RV EF as measured with cMRI. It also seems to lower diastolic blood pressure even when used for less than 4 h/night.

## Data Availability

Data is available.
