# Correction to: Effect of long term CPAP therapy on cardiac parameters assessed with cardiac MRI

**DOI:** 10.1007/s10554-021-02385-y

**Published:** 2022-01-29

**Authors:** W. Wuest, M. S. May, M. Wiesmueller, M. Uder, A. Schmid

**Affiliations:** Radioloical Institute, Friedrich-Alexander-University-Erlangen-Nuremberg, Maximiliansplatz 1, 91054 Erlangen, Germany

## Correction to: The International Journal of Cardiovascular Imaging (2021) 37:613–621 10.1007/s10554-020-02024-y

The article “Efect of long term CPAP therapy on cardiac parameters assessed with cardiac MRI written by W. Wuest, M. S. May, M. Wiesmueller, M. Uder and A. Schmid”, was originally published Online First without Open Access. After publication in volume 37, issue 2, page 613–621 the author decided to opt for Open Choice and to make the article an Open Access publication. Therefore, the copyright of the article has been changed to © The Author(s) 2021 and the article is forthwith distributed under a Creative Commons Attribution 4.0 International License, which permits use, sharing, adaptation, distribution and reproduction in any medium or format, as long as you give appropriate credit to the original author(s) and the source, provide a link to the Creative Commons licence, and indicate if changes were made. The images or other third party material in this article are included in the article’s Creative Commons licence, unless indicated otherwise in a credit line to the material. If material is not included in the article’s Creative Commons licence and your intended use is not permitted by statutory regulation or exceeds the permitted use, you will need to obtain permission directly from the copyright holder. To view a copy of this licence, visit https://creativecommons.org/licenses/by/4.0. Open Access funding enabled and organized by Projekt DEAL.

The original article has been corrected.

